# microRNA, the Innate-Immune System and SARS-CoV-2

**DOI:** 10.3389/fcimb.2022.887800

**Published:** 2022-06-16

**Authors:** James M. Hill, Walter J. Lukiw

**Affiliations:** ^1^ Louisiana State University (LSU) Neuroscience Center, Louisiana State University Health Science Center, New Orleans, LA, United States; ^2^ Department of Ophthalmology, LSU Health Science Center, New Orleans, LA, United States; ^3^ Department of Pharmacology, Louisiana State University (LSU) Health Science Center, New Orleans, LA, United States; ^4^ Department of Microbiology, Louisiana State University (LSU) Health Science Center, New Orleans, LA, United States; ^5^ Department Neurology, Louisiana State University Health Science Center, New Orleans, LA, United States

**Keywords:** Alzheimer’s disease, COVID-19, hsa-miRNA-15b-5p, hsa-miRNA-146a-5p, messenger RNA (mRNA), microRNA (miRNA), SARS-CoV-2, single-stranded viral RNA (ssvRNA)

## Abstract

The single-stranded viral RNA (ssvRNA) known as the severe acute respiratory syndrome coronavirus 2 (SARS-CoV-2) that causes COVID-19 can be effectively inactivated by a number of natural ribonucleic acid-based host cell defenses. One of the most important of these defenses includes the actions of a class of small non-coding RNAs (sncRNAs) known as microRNAs (miRNAs). *Via* base-pair complementarity miRNAs are capable of specifically targeting ssvRNA sequences such as SARS-CoV-2 promoting its inactivation and neutralization. RNA-sequencing and bioinformatics analysis indicate that multiple naturally-occurring human miRNAs have extensive complementarity to the SARS-CoV-2 ssvRNA genome. Since miRNA abundance, speciation, and complexity vary significantly amongst human individuals, this may in part explain the variability in the innate-immune and pathophysiological response of different individuals to SARS-CoV-2 and overall susceptibility to ssvRNA-mediated viral infection.

## OVERVIEW – SEVERE ACUTE RESPIRATORY SYNDROME CORONAVIRUS-2 (SARS-CoV-2)

Possessing an unusually large, positive-sense, ssvRNA genome of about ~29,903 nucleotides (nt; SARS-CoV-2 isolate Wuhan-Hu-1, Ke et al., 2020; Sah et al., 2020; Wu et al., 2020; Mousavizadeh and Ghasemi, 2021; National Center for Biological Information (NCBI) GenBank Accession No. NC_045512.2; last accessed 16 May 2022), SARS-CoV-2: (i) is a member of the genus *Betacoronavirus* in the family *Coronaviridae* that includes other common pathogenic human influenza-causing ssvRNA Coronaviruses (hCoV-OC43, HKU1 and 229E), SARS and MERS-CoV (Sah et al., 2020; Mousavizadeh and Ghasemi, 2021; Raghuvamsi et al., 2021); (ii) consists of a nucleocapsid core containing genomic ssvRNA within a lipoprotein envelope forming a ~100 nm diameter spherical virion particle (Ke et al., 2020); (iii) structurally resembles a ‘typical’ large messenger RNA (mRNA) possessing a 5′ methyl cap structure, a 3′ poly(A) tail and ~10-14 overlapping open reading frames (ORFs) with minimal spacer regions, encoding ~29 proteins, not all of which have been fully characterized (Ke et al., 2020; Sah et al., 2020; Raghuvamsi et al., 2021); (iv) possesses one of the largest ssvRNA genomes of all known ssvRNA viruses and a correspondingly huge target for potential sncRNA and miRNA interaction (Finkel et al., 2021; Mousavizadeh and Ghasemi, 2021; Pogue and Lukiw, 2021; Lukiw, 2022); (v) as an ssvRNA virus is representative of the most common type of emerging viral disease in humans; this appears to be attributable to the high mutation rate in RNA viruses (compared to DNA viruses) that possess extremely high mutation rates of up to 10**^6^** times higher than that of their hosts (Pachetti et al., 2020); and (vi) orchestrates a multipronged strategy to impede host protein synthesis including the accelerated degradation of host cytosolic cellular mRNAs, thus facilitating viral takeover of the host mRNA pool in infected cells (Finkel et al., 2021; Lukiw, 2022). The main structural proteins of SARS-CoV-2 include the envelope (‘E’), membrane (‘M’), nucleocapsid (‘N’), replicase (‘R’; an RNA dependent RNA polymerase or RdRp), surface spike (‘S’) protein and several accessory viral-encoded proteins (Ke et al., 2020; Finkel et al., 2021; Siniscalchi et al., 2021). The SARS-CoV-2 viral lipoprotein envelope is decorated with ‘E’, ‘M’, and ‘S’ proteins - the ‘S’ protein is a class 1 homo-trimeric viral fusion protein possessing distinctive ‘head’ and ‘stalk’ domains essential for host cell entry *via* the angiotensin converting enzyme 2 (ACE2) receptor (see below; Ke et al., 2020; Raghuvamsi et al., 2021; Lukiw, 2021). As discussed further below, using various miRNA-mRNA and miRNA-ssvRNA search algorithms, ‘*in silico*’ analysis and experimental validation, multiple naturally occurring human host miRNAs have been both predicted and verified to target several of these key SARS-CoV-2 genomic protein-encoding regions (Arisan et al., 2020; Finkel et al., 2021; Zhao et al., 2021). This Perspectives paper will address some of the most current findings and emerging concepts in this fascinating research area of potential miRNA contribution to human innate-immunity with special reference to natural host miRNAs, SARS-CoV-2, and the current COVID-19 pandemic.

## miRNA, Mechanism of Action and the Innate-Immune System

The discovery of the first microRNA (miRNA) *Lin-*4 in 1993 in the nematode *Caenorhabditis elegans* and its post-transcriptional targeting and down-regulation of the target mRNA encoded by the heterochronic developmental gene *Lin-14* revolutionized the entire field of molecular biology (Lee et al., 1993; Wightman et al., 1993; Bartel, 2018). It was not until almost a decade later that multiple miRNAs were first identified and characterized in *Homo sapien* cells and tissues (Lagos-Quintana et al., 2001; Hammond, 2015). About 15 years ago appeared the first reports on the interplay between host-derived miRNAs, inflammation and innate-immunity during health, viral infection, neurological disease and cancer (Calin and Croce, 2006; Lukiw, 2007; Bhela and Rouse, 2018; O'Brien et al., 2018; Mishra et al., 2020; Finkel et al., 2021; Padda et al., 2021). It has been established that the major mechanism of miRNA action is to bind *via* base-pair complementarity to single-stranded target mRNA, and in doing so, down-regulate or neutralize their biological activities (Hammond, 2015; Bartel, 2018; Lukiw, 2021). Taken together these studies uncovered complex, highly interactive and selective miRNA-viral and host-cell interactions. These included interplay among the pro-inflammatory- and innate-immune microRNA-146a (miRNA-146a), the neurotropic double-stranded DNA (dsDNA) herpes-simplex virus 1 and human neurons (HSV-1; Hill et al., 2009; Pogue and Lukiw, 2021). Largely based upon these original studies on direct miRNA-target mRNA interaction there is currently an expanding interest linking direct host miRNA interaction with ssvRNA species such as SARS-CoV-2 and subsequent viral inactivation, degradation and down-regulation of viral activity and capability for successful infection in the human host (Plotnikova et al., 2019; Mishra et al., 2020; Pierce et al., 2020; Wicik et al., 2020; Lukiw, 2021; Mousavizadeh and Ghasemi, 2021; Narożna and Rubiś, 2021; Okuyan and Begen, 2021). Current research further supports the concept that the actions of the 2,650 known ~20-24 nucleotide (nt) human miRNAs and other small non-coding RNAs (sncRNAs) and/or interfering RNAs (iRNAs) form the functional basis for a novel division of the host innate-immune system and its management of viral genomic activity that has implications for the efficiency of ssvRNA invasion and potential for infection.

## The Angiotensin Converting Enzyme 2 (ACE2) Receptor

The ACE2 receptor is a zinc-containing carboxypeptidase membrane-integral cell-surface receptor widely expressed in multiple cell types that is uniquely recognized by the SARS-CoV-2 virus ‘S’ glycoprotein for initial human host cell entry (Ke et al., 2020; Pierce et al., 2020; Hill et al., 2021; Lukiw, 2021; Raghuvamsi et al., 2021; Zhao et al., 2021). As the major SARS-CoV-2 cell surface-exposed transmembrane glycoprotein receptor ACE2 displays a significant variability in abundance on multiple human cell types and this may not only add another degree of variability for SARS-CoV-2 cellular invasion but also modulates SARS-CoV-2 exposure to the variable miRNA abundance and speciation in different human cell types (Ke et al., 2020; Hill et al., 2021; Zhao et al., 2021). In fact, the ACE2 receptor, the gateway for SARS-CoV-2 entry into the host cell, has a remarkable ubiquity, and has been detected on the surface of every human cell type so far analyzed with the exception of the erythrocyte, thus making it among the most prevalent receptor subtype encountered in all of human physiology (Hill et al., 2021; Jones et al., 2021; Zhao et al., 2021). Interestingly, the ACE2 receptor mRNA is itself targeted by multiple natural host miRNAs and this may also have a bearing on the availability of ACE2 receptor abundance on multiple cell and tissue types in different human hosts – an observation that may be useful in the personalized diagnosis of COVID-19 (Wicik et al., 2020). Together these findings support the concept: (i) that multiple cell types and tissues of the human respiratory, circulatory, cardiovascular, digestive and genitourinary system, hematic, lymphatic and glymphatic systems, and the central and peripheral nervous systems (CNS, PNS) provide multiple potential entry portals for SARS-CoV-2 invasion; and (ii) that the invasion of SARS-CoV-2 into multiple human cell types would also expose this ssvRNA to multiple cell-enriched or cell-specific species of miRNA. This may explain in part the wide and variable range of systemic involvement of SARS-CoV-2 infection and the wide spectrum of symptoms observed in COVID-19 patients. Indeed, while SARS-CoV-2 initially causes severe and acute respiratory distress and a highly lethal viral pneumonia it also presents with multiple ancillary complications involving multiple cell and tissue systems. An important example of focused SARS-CoV-2 attack is that within the normal human brain and CNS, extremely high levels of ACE2 receptor expression and abundance are found within the Botzinger complex of the rostral ventrolateral medulla, ventral respiratory column and other medullary respiratory centers of the brainstem, and this in part may explain the susceptibility of numerous COVID-19 patients to serious disturbances in quiet, restful breathing (eupnea) and the onset of severe respiratory complications (Hill et al., 2021; Mousavizadeh and Ghasemi, 2021; Lukiw, 2021; Raghuvamsi et al., 2021; Zhao et al., 2021). An increasing gradient of ACE2 receptor density in the human visual system may aid in the translocation of SARS-CoV-2 from the moist surface of the exterior of the eye into deeper regions of the visual brain (Lukiw, 2022). Another noteworthy example is that about one third of all COVID-19 patients experience neurological and/or neuropsychiatric symptoms, and a pre-existing diagnosis of Alzheimer’s disease (AD) predicts the highest risk for COVID-19 yet identified, especially among elderly AD patients. ACE2 expression and the density of ACE2 receptors have recently been found to be significantly up-regulated in the temporal lobe neocortex and hippocampal CA1 regions of AD-affected brain, anatomical regions targeted by the inflammatory neuropathology that characterizes AD, and this suggests a significant mechanistic overlap between AD and successful SARS-CoV-2 and other viral infections of the human CNS (Hill et al., 2009; Zhao et al., 2021; Choe et al., 2022; Lingor et al., 2022; Sirin et al., 2022; Szabo et al, 2022; Wang et al., 2022).

## Human microRNA and SARS-CoV-2 – Complexity by the Numbers

In *Homo sapiens* miRNAs possess very highly selected and ‘*evolutionary engineered’* ribonucleotide sequences and currently represent the smallest known information-carrying sncRNA sequences yet described. With regard to evolutionary selection, a fascinating and often overlooked fact is that a ~22 nucleotide miRNA with the possibility of 4 ribonucleotides (A,G,C or U) at each of the 22 positions could yield the staggering possibility of 4**^22^** (an exponentiation of four by the power of twenty-two) or about ~1.76x10**^13^** potential sncRNA sequences, however only about ~2.65 x10**^3^** miRNA sequences have been detected in all of human biology (miRBase ver 22.1, https://www.mirbase.org/; GENCODE data ver 38, https://www. gencodegenes.org/human/; last accessed 30 April 2022). This represents an extraordinary evolutionary selection pressure of just one ‘*biologically useful*’ miRNA out of every 6.8x10**^9^** prospective possible miRNA sequences that had potential to be generated. These ~2.65 x10**^3^** highly selected miRNAs that are present and function in human biology are currently believed to collectively regulate one third all of the genes in the human genome and are involved in the post-transcriptional control of gene expression patterns, the dynamic regulation of the cell’s transcriptome, neurodevelopment, aging and disease, including many human systemic disorders ranging from multiple types of cancer to Alzheimer’s disease (AD), other forms of age-related neurodegenerative disease and the modulation of the invasion of the host by pathogenic microbes (Lukiw, 2007; O'Brien et al., 2018; Plotnikova et al., 2019; Chakraborty et al., 2020; Fregeac et al., 2020; Padda et al., 2021; Pogue and Lukiw, 2021; Rybak-Wolf and Plass (2021).

Regarding this latter function, recent evidence further indicates that miRNAs play an important role in the complex interplay between viruses (containing both DNA and RNA genomes) and host cell genetics (Hill et al., 2009; Mishra et al., 2020; Wicik et al., 2020; Narożna and Rubiś, 2021; Schultz et al., 2021; Siniscalchi et al., 2021; Zhao et al., 2021). An interesting relevant example is that the NF-kB-sensitive *Homo sapiens* microRNA-146a (hsa-miRNA-146a-5p) is significantly over-expressed within brain and CNS tissues of progressive and often lethal viral-mediated neurological syndromes associated with age and advancing inflammatory neurodegeneration, and these include ~18 different viral-induced encephalopathies involving both single- and double-stranded RNA and/or DNA viruses (Pogue and Lukiw, 2021; Kucher et al., 2022). Despite huge research efforts, whether this represents part of the host’s adaptive immunity, innate-immune response or a mechanism to enable the invading virus a successful infection is currently not well understood (Jones et al., 2021; Narożna and Rubiz 2021; Pogue and Lukiw, 2021). Another specific and rather enigmatic example is that recent *in silico* analyses have determined that about ~600 of these host miRNAs, representing about one quarter of the total number of currently identified miRNAs in human cells have potential to interact with the SARS-CoV-2 genome (Siniscalchi et al., 2021; Ying et al., 2021; unpublished observations; see below). The RNA dynamics, sequence selectivity and complexity of this large family of potentially *‘anti-viral host miRNAs’* is certainly perplexing because their existence has long preceded the appearance and invasion of the SARS-CoV-2 virus into human populations. One explanation may be that conserved areas of the SARS-CoV-2 genome such as the ORF that encodes the SARS-CoV-2 viral replicase (RdRp) complex, consisting of a set of proteins required to produce infectious genomes, retain very highly conserved features in eukaryotic replicase-type enzymes and the RNA sequences that encode them over considerable periods of evolution (https://www.sciencedirect.com/topics/neuroscience/rna-viruses; last accessed 30 April 2022; Hillen et al., 2020; Pachetti et al., 2020).

## Anti-SARS-CoV-2 miRNA’s

It is well established that the major functions of host miRNAs include: (i) mRNA silencing involving miRNA-mediated repression of the expression of genetic information encoded in the target mRNA; (ii) the post-transcriptional regulation of gene expression patterns that extends into the shaping of the transcriptome of the cell during development and in health, aging and disease; and (iii) in playing a potential host-protective role in neutralizing microbial and ssvRNA invasion, including those by *Coronaviruses* such as SARS-CoV-2, thus contributing to a novel miRNA-facilitated innate-immune system for that host (Hammond, 2015; Trobaugh and Klimstra, 2017; Bartel, 2018; Plotnikova et al., 2019; Pierce et al., 2020; Wicik et al., 2020; Jones et al., 2021; Lukiw, 2021; Pogue and Lukiw, 2021; Siniscalchi et al., 2021). The two most important parameters for miRNAs to find their target ssRNAs, ssvRNAs or mRNAs are: **(i)** base-pair complementarity between the ssRNA, the ssvRNA or the mRNA ‘seed region’; and **(ii)** the thermodynamic stability of the miRNA-target RNA hybrid (ΔG or free energy of association **E_A_**) with values of **E_A_** of less than −20 kCal/mol between the miRNA and its ssRNA target being most highly favored (Lagos-Quintana et al., 2001; Bartel, 2018; Pierce et al., 2020; Jones et al., 2021; Lukiw, 2021; Siniscalchi et al., 2021). In general, depending on the stringency of RNA hybridization parameters and most favorable energies of association (E**_A_**), over the last ~2 years multiple possible binding sites have been predicted for miRNA binding to SARS-CoV-2 ssvRNA targets and multiple recent examples are next given here.

The Uysal-Onganer group first predicted seven miRNAs including miRNA-1307-3p, miRNA-1468-5p, miRNA-3611, miRNA-3691-3p, miRNA-3934-3p, miRNA-5197 and miRNA-8066 could strongly bind to the SARS-CoV-2 genome and linked these to host responses and virus pathogenicity-related KEGG pathways significant for comorbidities (Arisan et al., 2020). In a similar *in silico* study the McLellan group described 10 miRNAs expressed in SARS-CoV-2 target cells filtered according to databases and published data, and reported miRNA-18b-5p, miRNA-197-5p, miRNA-338-3p, miRNA-1273d, miRNA-3154, miRNA-3935-5p, miRNA-4436a, miRNA-4661-3p, miRNA-4761-5p and miRNA-5096 strongly targeted the ORF1a, ORF1b, ORF7a and ‘S’ regions of the SARS-CoV-2 genome (Hosseini Rad Sm and McLellan, 2020). Another recent *in silico* study using miRBase, MiRanda and Gene Set Enrichment Analysis (GSEA) software provided evidence: **(i)** that the miRNA-29 family had the most binding sites (N=11) on the SARS-CoV-2 ssRNA genome; and **(ii)** that the top human host miRNA candidates targeting the SARS-CoV-2 ssvRNA genome include those of the miRNA-16, miRNA-21, miRNA-29a/b, let-7b, let-7e, miRNA-122 and miRNA-146a microRNA families and others (Jafarinejad-Farsangi et al., 2020). These *in silico* prediction studies often display variable end results due to the settings for miRNA-target RNA base-pair complementarity algorithms and the thermodynamic stability of the miRNA-ssRNA hybrid itself. *In sili*co studies have most recently been integrated with experimental target validation to ascertain *bona fide* SARS-CoV-2 targeting and ssvRNA neutralization. For example, in one recent study RNAhybrid 2.2 and MirTarget analytical programs predicted between 857 and 2654 miRNA-SARS-CoV-2 pairings, respectively. About ~600 target sequences common to both analytical programs were subsequently filtered to select miRNAs expressed in respiratory cells of the lung (one natural site of SARS-CoV-2 infection) that revealed a perfect match to the seed region (nucleotides 2–8) of the hybridizing miRNA (Pierce et al., 2020; Siniscalchi et al., 2021). Experimental target validation using psiCheck-2 luciferase reporter plasmids transfected into the human lung cell line A549 indicated that several SARS-CoV-2 ssvRNA targets were experimentally validated including; importantly, a *Homo sapien* 22 nt, lung-enriched hsa-miRNA-15b-5p that targets and represses ‘S’ protein expression (Trobaugh and Klimstra, 2017; Siniscalchi et al., 2021; https://www.genecards.org/cgi-bin/carddisp.pl?gene =MIR15B; https://www.mirbase.org/cgi-bin/mirna_entry.pl?acc=MI0000438; last accessed 30 April 2022; see below; **Table 1**. Taken together these data suggest that the identified miRNAs should interact with ssvRNAs within infected cells, thus contributing to the regulation of SARS-CoV-2 gene expression, viability and capability for host-cell invasion.

**Table 1:** miRNAs predicted to interact with the ssvRNA of SARS-CoV-2.

microRNA	Reference
let-7b	Jafarinejad-Farsangi et al., 2020
let-7e	Jafarinejad-Farsangi et al., 2020
miRNA-15b-5p	Trobaugh and Klimstra, 2017; Siniscalchi et al., 2021
miRNA-16	Jafarinejad-Farsangi et al., 2020
miRNA-18b-5p	Hosseini Rad Sm and McLellan, 2020
miRNA-21	Jafarinejad-Farsangi et al., 2020
miRNA-29a/b	Jafarinejad-Farsangi et al., 2020
miRNA-122	Jafarinejad-Farsangi et al., 2020
miRNA-146a	Jafarinejad-Farsangi et al., 2020
miRNA-197-5p	Hosseini Rad Sm and McLellan, 2020
miRNA-338-3p	Hosseini Rad Sm and McLellan, 2020
miRNA-1273d	Hosseini Rad Sm and McLellan, 2020
miRNA-1307-3p	Arisan et al., 2020
miRNA-1468-5p	Arisan et al., 2020
miRNA-3154	Hosseini Rad Sm and McLellan, 2020
miRNA-3611	Arisan et al., 2020
miRNA-3691-3p	Arisan et al., 2020
miRNA-3934-3p	Arisan et al., 2020
miRNA-3935-5p	Hosseini Rad Sm and McLellan, 2020
miRNA-4436a	Hosseini Rad Sm and McLellan, 2020
miRNA-4661-3p	Hosseini Rad Sm and McLellan, 2020
miRNA-4761-5p	Hosseini Rad Sm and McLellan, 2020
miRNA-5096	Hosseini Rad Sm and McLellan, 2020
miRNA-5197	Arisan et al., 2020
miRNA-8066	Arisan et al., 2020

SARS-CoV-2 or other single-stranded viral RNAs (ssvRNAs) may be recognized (via base-pair complementarity) and degraded by miRNA-mediated interactions within the cell cytoplasm; there are multiple types of evidence that at least 25 miRNAs have potential to target SARS-CoV-2 and other ssvRNA sequences (Arisan et al., 2020; Hosseini Rad Sm and McLellan, 2020; Jafarinejad-Farsangi et al., 2020; Kucher et al., 2022); the natural functions of most of the miRNAs listed in **Table 1** are not known (see manuscript text); interestingly one recent in silico study using miRBase, MiRanda and Gene Set Enrichment Analysis (GSEA) software provided evidence: (i) that the miRNA-29 family had the most binding sites (N=11) on the SARS-CoV-2 ssvRNA genome (Jafarinejad-Farsangi et al., 2020); and (ii) using RNA-sequencing analysis, RNAhybrid 2.2 and MirTarget analytical programs between 857 and 2654 miRNAs have potential to interact with the ~29,903 nt SARS-CoV-2 ssvRNA genome (SARS-CoV-2 isolate Wuhan-Hu-1, National Center for Biological Information (NCBI) GenBank Accession No. NC_045512.2; last accessed 30 April 2022; Ke et al., 2020; Sah et al., 2020; Wu et al., 2020; Mousavizadeh and Ghasemi, 2021; Kucher et al., 2022). The SARS-CoV-2 ssvRNA genome thereby presents a potential target for naturally occurring human miRNA-mediated ssvRNA inactivation and neutralization; this may play an under-appreciated role in natural host immunity and the high variability in the innate-immune and pathophysiological response of different human individuals to SARS-CoV-2 and their overall susceptibility to ssvRNA-mediated viral infections that include COVID-19.

It should also be mentioned: (i) that both the ACE2 receptor and accessory ACE2-associated viral entry proteins such as the transmembrane serine protease 2 (TMPRSS2) and other cellular membrane ACE2-associated proteins may also have their encoding mRNAs targeted by specific sncRNAs and/or miRNAs which may further modulate the success of SARS-CoV-2 infectivity (Pierce et al., 2020; Wicik et al., 2020); and **(ii)** that the actions of host miRNAs on ssvRNAs are variable and depend on multiple endogenous and exogenous factors. While some host miRNAs may reinforce host antiviral responses against viruses by neutralizing ssvRNA sequences, some may also promote viral RNA stability, replication, and support successful infectivity (Trobaugh and Klimstra, 2017; Wicik et al., 2020). Another interesting consideration is that human population differences in individual miRNA abundance, speciation and complexity have been shown in different human and animal populations and in different cell- and tissue-types (Huang et al., 2011; He et al., 2017; Pogue and Lukiw, 2021). Different endogenous miRNA populations of human cells and tissues and the heterogeneous repertoire of different miRNAs in individual humans may in part explain: (i) the widely observed differential variability and human sensitivity and susceptibility to ssRNA viral infection such as SARS-CoV-2; (ii) the endogenous innate-immune capability to neutralize these and other invading microbial species; and (iii) the basis for a potential strategy in using stabilized miRNAs for the neutralization of SARS-CoV-2 and the therapeutic management of COVID-19 and other life-threatening microbial-mediated diseases (Lukiw, 2021; Padda et al., 2021; Ying et al., 2021).

## Conclusion

Emerging evidence continues to support the concept that as a novel form of immune surveillance, human miRNAs, sncRNAs and small iRNAs have a significant potential to shape the host’s innate-immune response to infection by invading ssvRNA viruses that include SARS-CoV-2. This ‘Perspectives’ paper proposes that the actions of the ~2,650 known human miRNAs constitute, in part, the basis for an under-recognized and under-appreciated innate-immune regulatory system for modulating ssvRNA viral genome activities that also has implications for the efficiency of SARS-CoV2 invasion, infectivity and viral replication. As miRNA abundance, speciation, and complexity varies significantly among human individuals, this may: (i) explain in part the variability in the innate-immune immunological and pathophysiological response of different human individuals to the initiation and progression of SARS-CoV-2 infection; and **(ii)** further support our understanding of the variable susceptibility and resistance of individuals to ssvRNA-mediated viral infection and COVID-19 and perhaps to other ssvRNA and related viral-mediated pandemics that may arise in the future on a global scale.

Lastly, evolving evidence continues to suggest that pathological ssvRNA genomes like SARS-CoV-2 are susceptible to attack, destruction, neutralization, and/or modulation by multiple naturally-occurring host miRNAs. While the basic mechanisms of miRNA-ssvRNA natural hybrid formation and selection are becoming increasingly understood, the specific interaction of human host miRNA with ssvRNAs like SARS-CoV-2 in human physiology remains extremely complicated and perplexing. Cellular, transcriptional, genetic, epigenetic, immunological, metabolic, developmental, environmental and other epidemiological conditions affect the generation and processing of host miRNAs leading to dynamic, temporal and cell- and tissue-specific patterns of miRNA abundance, speciation and complexity (O'Brien et al., 2018; Pogue and Lukiw, 2021; Wicik et al., 2020; Ying et al., 2021). There are also aging-, gender- and disease-associated effects on miRNA expression patterns in human biology. For example, some ‘beneficial’ human anti-viral miRNAs naturally down-regulated with aging may display modified SARS-CoV-2‐host cell interactions that enhance the severity and mortality among elderly COVID-19 patients, particularly those greater than 65 years of age (Fulzele et al., 2020; Choe et al., 2022; Kucher et al., 2022; Lingor et al., 2022). When compared to normal cells and tissues, diseases such as cancer and AD display different intrinsic patterns of host miRNA expression throughout these disease processes. Inter-current SARS-CoV-2 infection with these and other life-threatening/incapacitating diseases usually predict an unfavorable clinical outcome. One of the initial miRNA-based pharmaceuticals which specifically targets and down-regulates ssvRNA levels (*Miravirsen*; Santaris Pharma A/S/Hoffmann-La Roche-Genentech, San Francisco USA) is currently in Phase III clinical trials and there is emerging and supportive evidence that stabilized miRNAs have strong potential to down-regulate and/or neutralize viral replication, modulate the progress of viral infection and/or enhance survival rates especially in the more advanced and severely-affected COVID-19 patients (Chakraborty et al., 2020; Narożna and Rubiś, 2021; Okuyan and Begen, 2021; Schultz et al., 2021; Choe et al., 2022; Kucher et al., 2022).

## Data Availability Statement

The original contributions presented in the study are included in the article/supplementary material. Further inquiries can be directed to the corresponding author.

## Author Contributions

WL and JH conceptualized this perspective article and all authors contributed to the writing, reading and approval of the final manuscript.

## Funding

This research was funded by an unrestricted grant from the LSU Eye Center Research to Prevent Blindness (RPB), the Brown Foundation, Joe and Dorothy Dorsett Innovation in Science Healthy Aging Award, the Louisiana Biotechnology Research Network (LBRN) and National Institute of Health (NIH) grants NEI EY006311, NIA AG18031 and NIA AG038834 (WL).

## Conflict of Interest

The authors declare that the research was conducted in the absence of any commercial or financial relationships that could be construed as a potential conflict of interest.

## Publisher’s Note

All claims expressed in this article are solely those of the authors and do not necessarily represent those of their affiliated organizations, or those of the publisher, the editors and the reviewers. Any product that may be evaluated in this article, or claim that may be made by its manufacturer, is not guaranteed or endorsed by the publisher.

## Abbreviations

COVID-19, coronavirus disease of the year 2019; has, *Homo sapien*; KEGG, Kyoto encyclopedia of genes and genomes; mRNA, messenger RNA (mRNA); miRNA, microRNA; RdRp, RNA dependent RNA polymerase; SARS-CoV-2, severe acute respiratory syndrome coronavirus-2; single-stranded viral RNA, (ssvRNA); WHO, World Health Organization.
